# Mitigation of Hepatic Impairment with Polysaccharides from Red Alga *Albidum corallinum* Supplementation through Promoting the Lipid Profile and Liver Homeostasis in Tebuconazole-Exposed Rats

**DOI:** 10.3390/ph16091305

**Published:** 2023-09-15

**Authors:** Hajer Ben Saad, Donyez Frikha, Amir Bouallegue, Riadh Badraoui, Manel Mellouli, Hatem Kallel, Jean Marc Pujo, Ibtissem Ben Amara

**Affiliations:** 1 Laboratory of Medicinal and Environment Chemistry, Higher Institute of Biotechnology, University of Sfax, Sfax 3000, Tunisia; 2 Laboratory of Marine Biodiversity and Environment, University of Sfax, Sfax 3000, Tunisia; 3 Laboratory for the Improvement of Plants and Valorization of Agroressources, National School of Engineering of Sfax, Sfax 3038, Tunisia; 4 Laboratory of General Biology, University of Ha’il, Ha’il 45851, Saudi Arabia; 5 Section of Histology-Cytology, Medicine Faculty of Tunis, University of Tunis El Manar, Tunis 1007, Tunisia; 6 Anatomopathology Laboratory, University of Sfax, Habib Bourguiba Hospital, Sfax 3029, Tunisia; 7 Intensive Care Unit, Cayenne General Hospital, Cayenne 97300, French Guiana; 8 Tropical Biome and Immunopathology, Inserm U 1019, University of Guyane, Cayenne 97346, French Guiana; 9 Emergency Department, Cayenne General Hospital, Cayenne 97300, French Guiana

**Keywords:** *Alsidium corallinum*, polysaccharides, biological activities, hepatotoxicity, dyslipidemia, computational analysis

## Abstract

Sulfated polysaccharides from seaweed are highly active natural substances with valuable applications. In the present paper, attempts have been made to discuss the physicochemical and structural features of polysaccharides isolated from red marine alga *Alsidium corallinum* (ACPs) and their protective effect in hepatic impairments induced by tebuconazole (TEB) in male adult rats. Structural features were determined using high-performance liquid chromatography, Fourier-transformed infrared, and solid-state ^1^H and ^13^C-Nuclear magnetic resonance analysis. ACPs are found to be hetero-sulfated-anionic polysaccharides that contain carbohydrates, sulfate groups, and uronic acids. In vitro biological activities suggested the effective antioxidant and antimicrobial capacities of ACPs. For antioxidant testing in vivo, the biochemical analysis and plasma profiles displayed that oral administration of ACPs could mitigate blood lipid indicators, including total cholesterol, triglyceride, low and high-density lipoprotein cholesterol, and bilirubin. Liver function indexes involving alanine aminotransferase and aspartate aminotransferase showed that ACPs possessed prominent antioxidant activities. Additionally, the intervention of ACPs potentially inhibited lipid peroxidation, protein oxidation, key enzymes of lipid metabolism (<0.001), and improved antioxidant status (<0.05). Histomorphological observation confirmed that ACPs intervention could partially repair liver injuries caused by TEB. The computational results showed that *A. corallinum* monosaccharides bound 1JIJ, 1HD2, and 1WL4 receptors with acceptable affinities, which, together with deep embedding and molecular interactions, support the antioxidant, antimicrobial, and hypolipidemic outlined effects in the in vitro and in vivo findings. Given their prominent antioxidant effects, ACPs are promising candidates for liver diseases and must be considered in pharmaceutical applications.

## 1. Introduction

Increasing interest in healthy human food worldwide introduces a rising consumption of natural compounds [1], which are known for their various biological activities and have been applied for a long time in the pharmaceutical area. They are often characterized as multi-component and multi-functional to treat diseases, which might be more effective due to possible synergistic actions [2].

Marine algae have recently been considered a new source of bioactive compounds [3]. Polysaccharides are the most common macromolecules produced by algae [4,5]. Like polynucleotides and proteins, polysaccharides play key roles in life activities such as fertilization, signal recognition, cell communication and adhesion, blood clotting, pathogenesis prevention, and system development [6,7,8]. Recently, pharmacological reports have shown that polysaccharides isolated from algae have various pharmacological activities, including immunomodulation, antitumor, anti-inflammatory, and antioxidant capacities [7,8]. The biological abilities of polysaccharides are probably related to the presence and positions of sulfate groups. The macromolecules have different levels of spatial organization, including primary, secondary, tertiary, and quaternary structures. Their primary forms can affect the various properties of polysaccharides, like their water-solubility, gel viscosity, and biological functions [9]. Functional polysaccharides could prevent the oxidative stress provoked by reactive oxygen species (ROS).

Generally, the endogenous antioxidant system can scavenge ROS amounts generated by cellular metabolism [10]. However, excessive environmental stresses, like ultraviolet irradiation and toxic chemicals, such as pesticides, can cause abnormal ROS production, which leads to oxidative stress.

Tebuconazole (TEB) is an effective fungicide used for the control of mildew and rust on wheat, barley, rice, fruits, and vegetables [11]. In the long term, it was one kind of toxicant for marine organisms, as it might induce an adverse effect on the aquatic environment [12]. Animals were sensitive to the influence of TEB because they could uptake and retain xenobiotics from circumstances via active or passive processes. TEB provokes adrenal gland hypertrophy in chronic dog studies and mouse teratogenic effects [13,14]. Sancho et al. [15] showed that short-term exposure to TEB induced physiological impairment and endocrine reproduction perturbation in male zebrafish. TEB is also deemed to cause hepatic and reproductive damage by inducing lipid peroxidation, decreasing antioxidant enzyme activities, and releasing free radicals [12,15]. To date, no investigation has been carried out on the biological activities of polysaccharides obtained from *Alsidium corallinum (A. corallinum)*. Only a few studies have been realized on phenolic compounds extracted from *A. corallinum*, a red alga from the Mediterranean Sea, demonstrating their interesting biological activities, including antioxidant and hepatoprotective properties [16].

The present study aims to investigate the protective effect of ACPs against TEB-induced hepatotoxicity in adult rats. Identification of ACPs in vitro is necessary to understand their structural and functional properties and antioxidant potential. Furthermore, the binding affinities and molecular interactions of ACPs with TyrRS from *S. aureus* (1JIJ), human peroxiredoxin (1HD2), and Acyl-CoA: cholesterol acyltransferase (ACAT, 1WL4) were assessed using computational modeling.

## 2. Results and Discussion

### 2.1. Extraction Yield and Physicochemical Analysis of ACPs

The yield and chemical analysis of polysaccharides isolated from the alga *Alsidium corallinum* are reported in Table 1.

The yield percent of ACPs (20.93%) is better than the yields previously reported for polysaccharides from several other algae, including *Gelidium crinale* (2.6%) [17], *Sargassum swartzii* (11%) [18], and *Chaetomorpha linum* (16%) [19]. The yield is similar to polysaccharides isolated from fenugreek [20].

Polysaccharides are polar macromolecules that are easy to dissolve in water because they can replace water–water interactions with water–solute interactions. Polysaccharide extraction yield is influenced by algal species, period of collection, and extraction parameters. Moreover, the pH presented a 6.2 ± 03. Ktari et al. [21] reported that the pH of fenugreek polysaccharides solution at 37 °C is 6.4. ACPs have relatively low levels of moisture (2.96 ± 0.09%) and ash (3.00 ± 0.08%).

The quantitative estimation of ACPs showed a significant contribution of carbohydrates and less uronic acid and proteins. Proteins are part of cell wall structure, and are associated closely with polysaccharides. It has been considered a potential contaminant of polysaccharides [22]. After depigmentation and extraction of ACPs, we tried to denature proteins and eliminate the majority of lipids, and that is why we have acquired relatively low levels of proteins (3%) compared to other work done on the other polysaccharides [8], which suggests the efficiency of the extraction method. Amounts of proteins depend mainly on the way of extraction and deproteination processes. Fleury and Lahaye [23] indicated that precipitation of proteins during extraction at 100 °C contributed probably to their indigestibility. The homogeneity of the polysaccharide was confirmed by elemental microanalyses, which showed a protein content of 3% of protein residues. The ACPs protein contents were similar to those from the endodermis of Shaddock [24]. Our results indicate that extracting and isolating ACPs was suitable for yielding a compound free of undesired molecules that could interfere in the subsequent experiments.

Moreover, the results presented in Table 1 revealed that ACPs had relatively high carbohydrate levels (66.06%).

On the other hand, the uronic acid content of ACPs (11.03%) was similar to those obtained by polysaccharides extracted from *Sargassum vulgare* (brown alga) [25]. The marine origin, seasonal periods, conditions, and extraction method are determining factors for the variations in all these contents.

### 2.2. Spectroscopic Analysis of ACPs

#### 2.2.1. Monosaccharides by HPLC-FID

**Figure 1:**
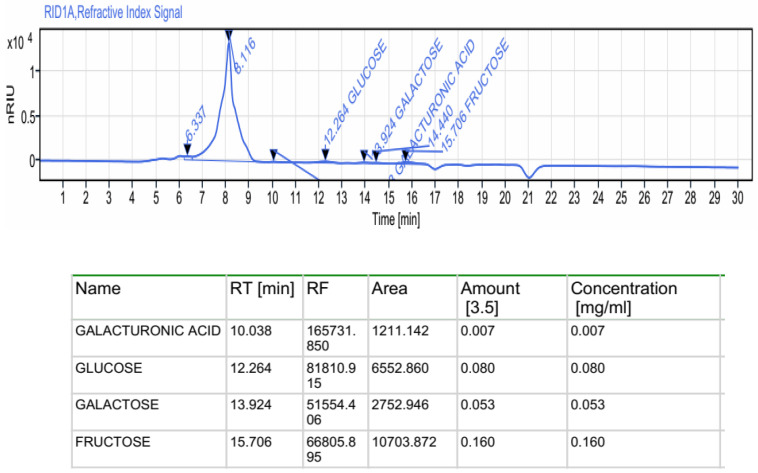
Monosaccharides composition of ACPs performed by HPLC-FID.

High-performance liquid chromatography (HPLC) analysis indicated heterogeneous composition in which galacturonic acid, glucose, galactose, and fructose are the major monosaccharide units at retention times of 10.038, 12.264, 13.924, and 15.607 min, respectively, according to the elution time a of monosaccharide standards (Figure 1). Previous data regarding polysaccharides extracted from macroalgae also revealed heterogeneous compositions of monosaccharides [26,27]. Robic et al. [28] have reported that the sugar contents can be attributed to the species, the collection period, and the sample extraction conditions.

#### 2.2.2. Nuclear Magnetic Resonance (NMR) Spectroscopy

**Figure 2:**
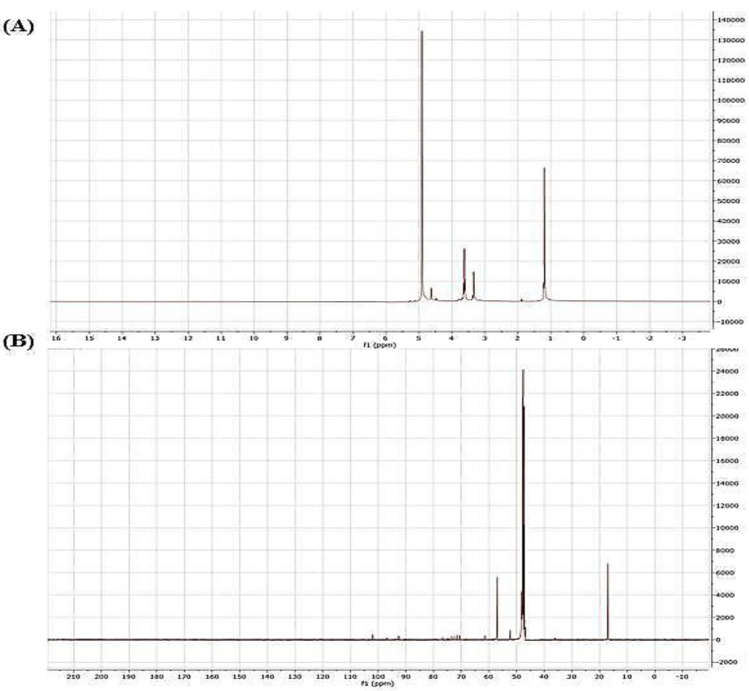
(**A**) ^1^H NMR and (**B**) solid state ^13^C NMR spectra of ACPs.

To further determine the sequential residue linkage of ACPs, the NMR data of ACPs were performed, as shown in Figure 2 (in liquid and solid-state NMR). Figure 2A matched the anomeric proton signals (δ1; 3.2; 3.5, and 5 ppm) and the anomeric carbon signal (δ 19, 49, and 58 ppm). The ^1^H NMR spectrum of the ACPs exhibited a set of wide and intense signals (3.0–4.0 ppm) due to CH2-O and CH-O groups of sugars. It also showed signals between 3.2 and 3.5 ppm associated with anomeric protons. Other more intense signals (5 ppm) are related to the intense signal of the solvent and are due to the anomeric protons [29].

The ^13^C NMR spectrum of ACPs in a solid state presented broad signals limiting its resolution. It showed, among others, a very intense signal at 50 ppm, certainly enveloping several peaks related to osidic CH2-O and CH-O groups. Less intense signals between 15.0 and 18.7 ppm could be assigned to the axial methyl resonates in sugars [30]. In addition, monosaccharides such as glucose, galacturonic acid, and glucuronic acid also exist in ACPs, but in minor amounts that were not easy to detect after methylation.

#### 2.2.3. Infra-Red Spectroscopic Analysis

**Figure 3:**
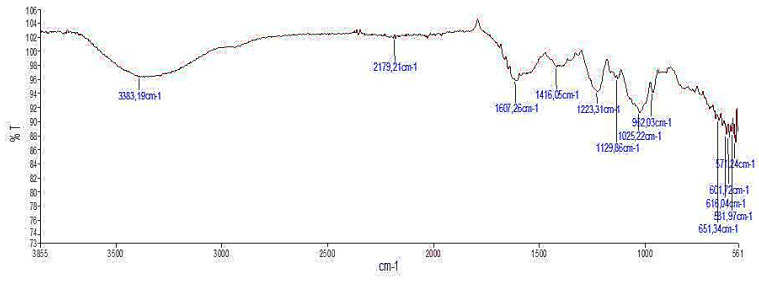
FT-IR spectra of ACPs.

The FT-IR spectrum of ACPs is shown in Figure 3, in which different absorption bands emerged following wave numbers. The FT-IR spectrum of ACPs displayed typical signals of polysaccharides. ACPs showed bands at 3383.19; 2179.21; 1607.26; 1426.06; 1223.31; 1129.88; 1025.22 and 962.03 cm^−1^. The broad band at 3383.19 cm^−1^ was due to the stretching vibration of O-H and N-H. Bands in the region of 2179 cm^−1^ represented the C-H stretching and bending vibration. The absorption peak at 1607 cm^−1^ was ascribed to C-O stretching vibration, which characterized uronic acid. The band at 1223 cm^−1^ was assigned to O=S=O stretching vibration of sulfate esters [31]. The adjacent peaks at 1129 cm^−1^ and 1025 cm^−1^ indicated the C-O-C and C-O-H stretching vibration, which demonstrated the presence of a pyranose ring [32]. The peak at 962 cm^−1^ was from the C-O-C vibration, suggesting that the sulfate group was mainly at C-3 position [33]. Based on the FT-IR spectrum, it could be anticipated that the ACPs may be predominantly acidic (Figure 3).

### 2.3. In Vitro Biological Activities of ACPs

Antioxidant activities of ACPs have been determined by various methods such as DPPH radical scavenging, ABTS radical scavenging, ferric-reducing antioxidant power, and nitric oxide scavenging assays.

#### 2.3.1. Effect of ACPs on DPPH Scavenging Activity

**Figure 4:**
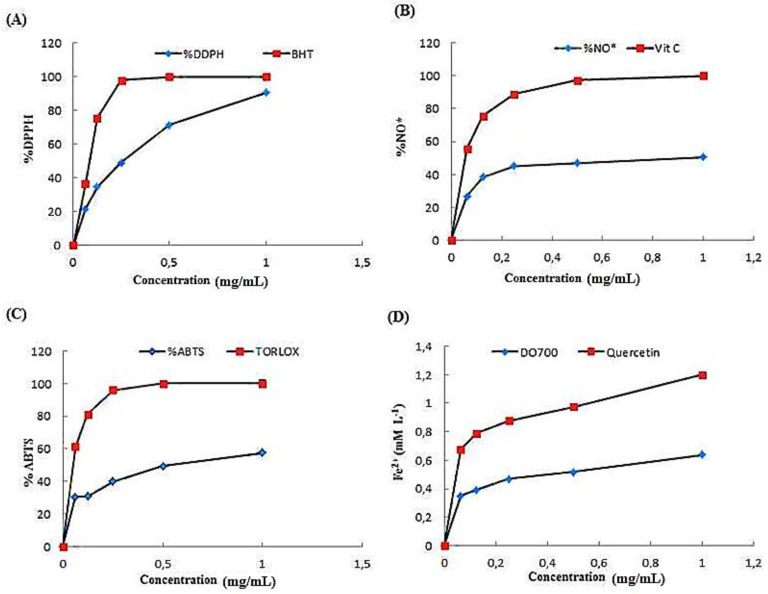
(**A**) DPPH radical-scavenging activity of ACPs; (**B**) metal ion-chelating activity of ACPs; (**C**) reducing power activity of ACPs; (**D**) ferric reducing antioxidant power of ACPs.

The DPPH scavenging activities of ACPs were presented in Figure 4A. Results indicated that ACPs exhibited high antioxidant activity against DPPH under a concentration-dependent approach. A maximum DPPH scavenging activity of ACPs at 1 mg/mL concentration with a percentage inhibition of 80% was observed. In contrast, BHT showed a maximum percentage inhibition of 100% at 0.25 mg/mL. Our results are similar to previous work by Khaskheli et al. [34], who reported a positive correlation between the DPPH scavenging activity and sample concentrations. The effects of ACPs on DPPH radical scavenging are thought to be due to their hydrogen-donating ability and sulfate group [35]. Furthermore, it has been reported that polysaccharides’ antioxidant activities were related to their sulfation degree, uronic acid content, molecular weight, and glycosidic linkage [36].

#### 2.3.2. Scavenging Effect of ACPs on Nitric Oxide Radical

ACPs showed maximum nitric oxide scavenging activity at 1 mg/mL with an inhibition of 40%. Vitamin C showed a maximum nitric oxide scavenging (100%) at 0.5 mg/mL (Figure 4B). Nitric Oxide (NO) is a diffusible free radical, which plays an essential role as an effector molecule in diverse biological systems such as antioxidant, antitumor, and antimicrobial activities [37]. According to Kim et al. [25], the polysaccharides obtained from *Sargassum fulvellum* are more potent NO scavengers than synthetic antioxidants, including α–tocopherol and BHA.

#### 2.3.3. ABTS Radical Scavenging Activity of ACPs

The total antioxidant ability of ACPs was measured through scavenging a protonated radical ABTS and compared with the Trolox standard. The present study results showed that ACPs reacted on ABTS radicals at different concentrations ranging from 0 to 1 mg/mL, respectively (Figure 4C).

ABTS radical was scavenged at a maximum concentration of 1 mg/mL with a percentage inhibition of 58% by ACPs, whereas Trolox showed 100% at 0.25 mg/mL. The scavenging ability of ACPs on ABTS free radicals demonstrated that the activities of every sample increased as the concentration increased. A positive correlation has been demonstrated between sulfate content and ABTS activity in polysaccharide fractions extracted from brown seaweed *Laminaria japonica* [38].

#### 2.3.4. Ferric Reducing Antioxidant Power (FRAP)

The direct correlation between reducing powers and antioxidant activities of bioactive compounds have been signaled previously [7,8]. In the present study, the capacity of ACPs to reduce Fe^3+^ to Fe^2+^ was determined. As indicated in Figure 4D, the reducing activity of ACPs and quercetin increased with increasing concentration. At the concentration of 1 mg mL^−1^, the FRAP value of ACPs was 0.6 mM L^−1^, which was weaker than that of quercetin (1.2 mM L^−1^). According to Ruperez et al. [35], the polysaccharides extracted from *Fucusve siculosus* have demonstrated for their antioxidant capacity using the FRAP assay.

#### 2.3.5. Antimicrobial Activity of ACPs

**Figure 5:**
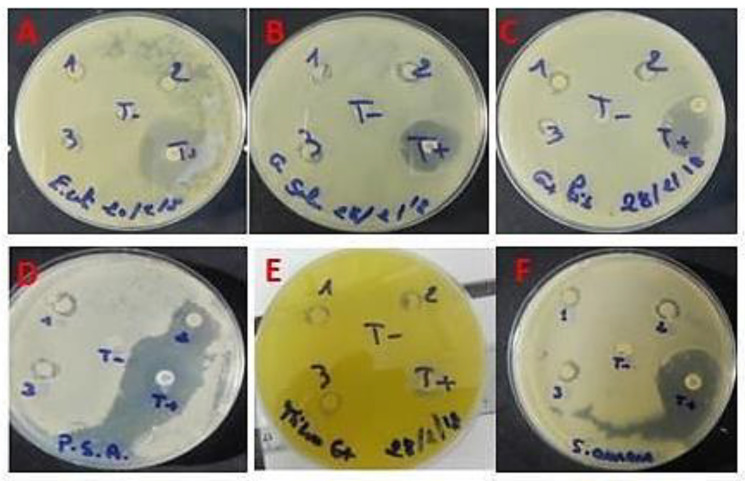
Diffusion test on agar. (**A**) *E. coli*, (**B**) *S. enterica*, (**C**) *L. invanovii,* (**D**) *P. aeruginosa,* (**E**) *M. luteus*, and (**F**) *S. aureus*.

The rise of antibiotic resistance remains a significant concern for pathogenic bacteria infections [39]. The seaweeds are of great interest given their antimicrobial properties [40]. Here, the disc diffusion method evaluated the new polysaccharides from *Alsidium corallinum* for their antimicrobial potential against six Gram (+) and Gram (−). The results reveal that the polysaccharides studied exert a considerable antibacterial effect on all the strains with inhibition zones of 8 mm and 10 mm, respectively, compared to the standard antimicrobial agent, ampicillin. Depending on the extent of the inhibition zone (Figure 5), we can classify ACPs in one of the following categories: Sensitive, Intermediate, or Resistant. CMB corresponds to the lowest extract concentration capable of killing 99.9% of bacteria in the starting inoculum. The results show that the values of CMI and CMB agree with those of the inhibition diameters. ACPs induced an important zone of inhibition exhibiting the lowest values of MIC on the corresponding strains (*E. coli*, *S. enterica*, *P. aeruginosa*, *M. lutus*, *L. invanovii*, and *S. aureus*).

Consequently, we compared the MIC and CMB of ACPs on the strains tested. The ACPs fractions show a bactericidal effect since the MIC/CMB ratios for all the target test strains of Gram^−^ and Gram^+^ are equal to 1 and/or 2 (Table 2). Our results support previous findings that Gram (+) bacteria are more sensitive to marine polysaccharide extracts than Gram (−) bacteria [41].

### 2.4. Phytotoxicity Essay of ACPs

**Figure 6:**
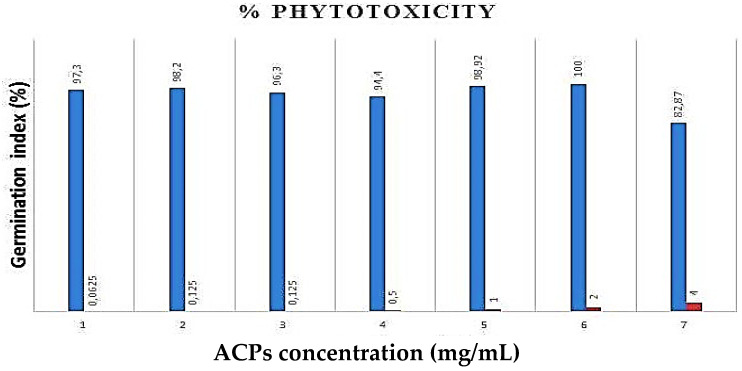
Effects of ACPs on the seed germination (%).

As shown in Figure 6, ACPs phytotoxicity was determined in different concentrations (1, 0.5, 0.25, 0.125, 0.0625 mg/mL) against cress seeds. Our results have shown that seed germination percent ≥80% with the different concentrations of ACPs, which confirms that polysaccharides from *Alsidium corallinum* are not phytotoxic [42].

### 2.5. In Vivo Biological Activities of ACPs

#### 2.5.1. Effect of ACPs on Toxicity Biomarkers in Hepatic Tissue

The liver is a vital organ responsible for drugs and toxic chemical metabolisms. Nearly all toxic chemicals frequently damage it, and its damage is considered a global health problem [43].

Lipid peroxidation is one of the most frequent reactions of free radicals’ attacks on biological structures. Extensive lipid peroxidation in cellular membranes caused disturbances of structural integrity, a decrease in membrane potential, increased permeability to ions, and a loss of fluidity [44]. Table 3 represents the level of MDA in the liver tissue homogenate of rats treated by TEB and ACPs. The TEB-treated rats exhibited a significant increase in hepatic MDA concentration compared to the control group (Table 3). An elevation of lipid peroxidation in animals treated with pesticide has been previously reported [45].

Lipid peroxidation can damage the hepatocytes membrane by accumulation of ROS. Cellular capacity to produce ROS has been generally evaluated by determining H_2_O_2_ levels released by mitochondrial respiration. TEB generated hepatic oxygen radical production through the increase in H_2_O_2_ radical levels.

Moreover, increased free radical generation can also lead to protein modifications. Locckie et al. [46] have reported that AOPPs can reflect an excess of free radical generation, confirming the protein oxidation. In our study, ROS, probably generated by TEB treatment, induced a rise of AOPPs in the liver of adult rats. However, ACPs administration alleviated lipid peroxidation and protein oxidation, probably via their antioxidant ability, emphasized through in vitro experiments. Thus, the natural polysaccharides can act as a defensive mechanism for antioxidant status in liver tissue, preventing the uncontrolled formation of ROS and/or directly scavenging the free radicals.

#### 2.5.2. Effect of ACPs on the Antioxidant Statute in Liver Tissue

Among the antioxidant enzymes, GSH-Px and SOD are the first-line defenses against oxidative stress injury and ROS generation [45]. Antioxidant enzyme activities in experimental animals are given in Table 4. TEB treatment significantly reduced (*p* < 0.05) the antioxidant defense of adult rats compared to controls. Reducing the antioxidant enzyme activities is the principal factor in lipid peroxidation damages [47]. A decrease in SOD and GSH-Px is due to the generation of ROS, leading to harmful effects such as loss of integrity and function of cell membranes [48]. Consistent with the earlier study, decreased hepatic SOD and GSH-Px activities were shown in the TEB-administered rats [49].

In contrast, TEB + ACPs and ACPs groups showed a significant increase (*p* < 0.05) in antioxidant activities compared to TEB group. Therefore, the increase in GSH-Px and SOD activities might be devoted to the antioxidant ability of ACPs in vitro. Zhang et al. [50] have shown an increase in antioxidant defense in aging mice after supplementation with polysaccharides fraction from *Porphyra haitanesis*. In addition, *Astragalus* polysaccharides exhibited hepatoprotective effects due to their capacity to scavenge free radicals, reduce oxidative stress, and inhibit lipid peroxidation [51]. Earlier reports have shown that the mechanism involved in the antioxidant activity is the ability of a molecule to donate a hydrogen atom to a radical, with the propensity of hydrogen donation being a critical factor [52,53]. Thus, hydrogen atom donors can terminate chain reactions by converting free radicals to more stable products. It has been hypothesized that the antioxidant capacity of polysaccharides is related to their molecular weight and chemical composition [36].

#### 2.5.3. Effect of ACPs on Liver Biochemical Markers

**Figure 7:**
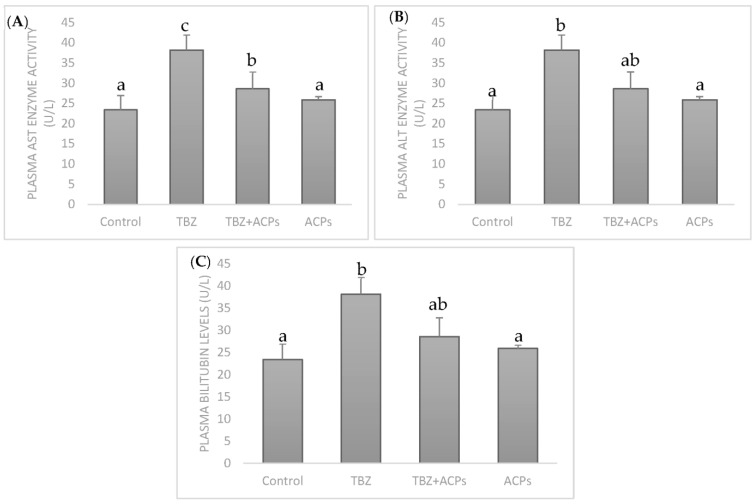
Effects of ACPs on liver function enzymes. (**A**) plasma AST activity, (**B**) plasma ALT activity, and (**C**) plasma bilirubin levels. Results are expressed as the means of three experiments ± SD. The number of determinations was n = 3. ^a,b,c^ In the same column indicate significant differences (*p* < 0.05).

The toxicological studies of TEB have been conducted by measuring plasma AST and ALT enzyme activities and bilirubin levels. The increased levels of these enzymes are indicators of liver damage. These are the most frequent indicators of necrosis of hepatic cells. AST is presented in the cytosol and mitochondria of tissues like skeletal muscle, heart, brain, liver, and kidney. ALT is localized in the cytosol of hepatocytes. Bilirubin is present in bile and formed as a by-product during the liver’s breakdown of old red blood cells [54]. Common biochemical markers of liver damage are the increase in ALT, AST, and bilirubin activities in the blood [55]. TEB resulted in increased plasma AST and ALT activities and bilirubin levels (Figure 7). Interestingly, the rats supplemented with ACPs reduced the abnormal changes significantly and recovered the activities of these enzymes to normal levels, displaying a meaningful result that the supplementation of ACPs has a protective effect against hepatotoxicity produced by TEB and could ameliorate the liver injury in TEB-treated rats, showing its potential to maintain the normal functional status of the liver, as reported by Lekshmi et al. [56] using the edible marine alga *Padina tetrastromatica*.

#### 2.5.4. Effect of ACPs on Plasma Lipid Levels

**Figure 8:**
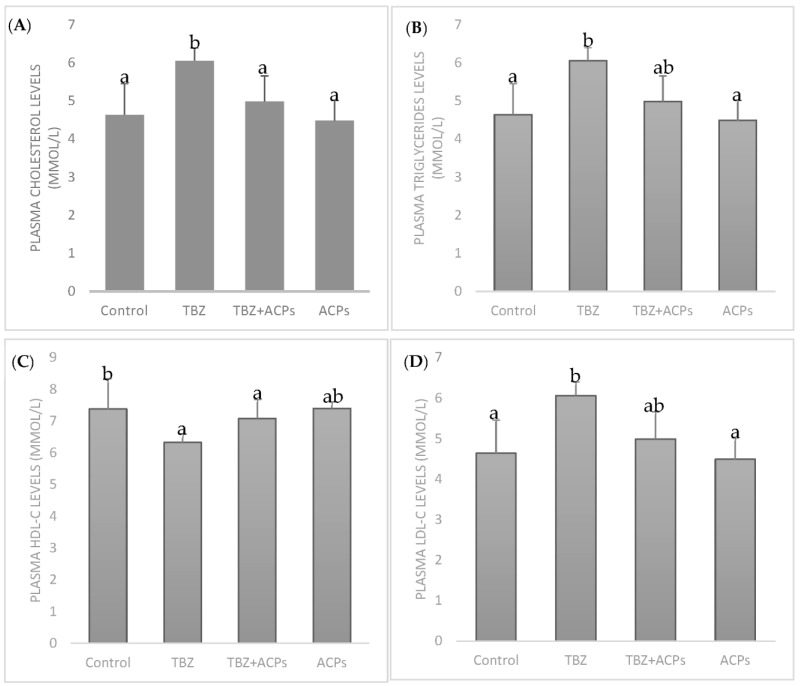
Effects of ACPs on plasma lipid profile. (**A**) Total cholesterol levels, (**B**) triglyceride levels, (**C**) HDL-C levels, and (**D**) LDL-C levels. Results are expressed as the means of three experiments ± SD. The number of determinations was n = 3. ^a,b^ In the same column indicate significant differences (*p* < 0.05).

After treatment for 4 weeks, the levels of TC, TG, and LDL-C in the plasma of the TEB-treated group were significantly higher than that of the control group. The HDL-C value in TEB model group significantly lowered. There was a positive correlation between plasma triacylglycerol, total cholesterol levels, and free radicals generation [56]. One of the harms of TEB is the accumulation of liver fat due to the metabolism disorders of blood lipids, which weakens the liver’s detoxification ability. However, all TC, TG, and LDL-C levels were significantly decreased by ACPs supplementation (Figure 8), while the HDL-C value increased significantly. All the levels recovered to a similar control group level, indicating ACPs can be a promising alternative for reducing plasma lipid levels. The results may be associated with its potential protection effect on hepatic cells by gearing up the detoxification machinery. It is believed that polysaccharides have shown anti-lipid effects in experimental research [57]. However, some chemical groups may play a key role in polysaccharide activities. Sulfate is one of these, closely associated with antioxidant activity [58]. The results of the present study indicate that ACPs ameliorate TEB-induced hyperlipidemia. With previous studies’ results, polysaccharides may be beneficial against hyperlipidemia and may be a suitable alternative hypolipidemic source for humans. These polymers were shown to have significant preventive effects on the elevation of cholesterol and triglycerides in plasma.

#### 2.5.5. Histopathological Analysis of Liver Tissue

**Figure 9:**
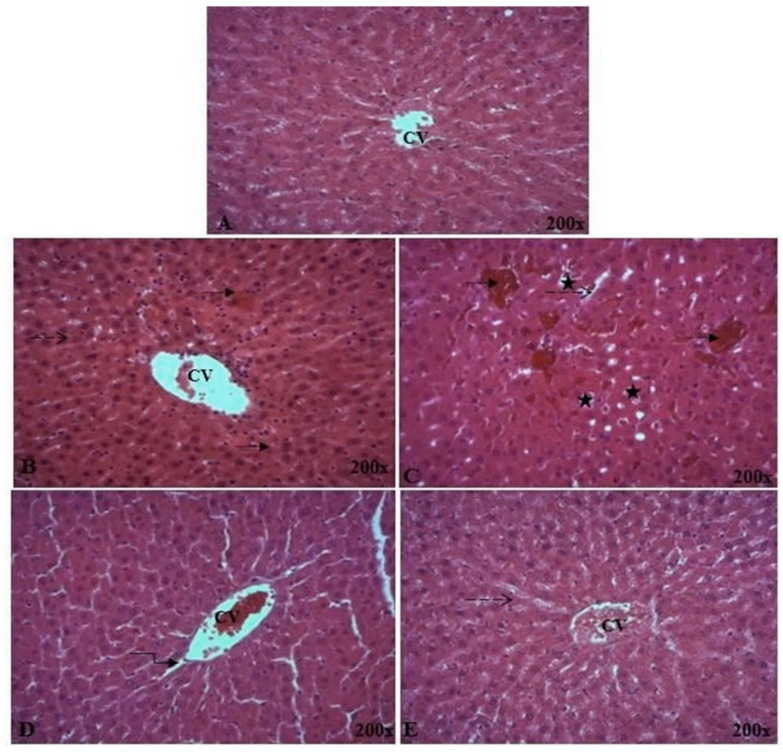

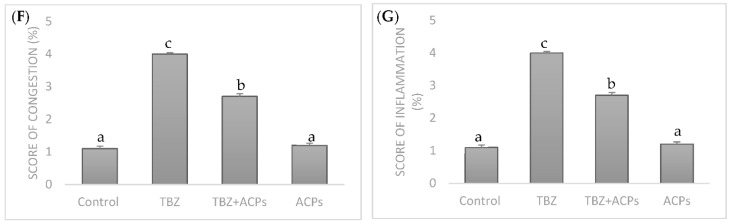
Light microscopic photographs of the liver’s paraffin section stained with H&E taken at 200× magnifications. (**A**) Control; (**B**,**C**) TEB; (**D**) TEB + ACPs; (**E**) ACPs, (**F**,**G**) histological NAS scores of liver tissue. Arrows indicate: 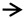 vascular congestion; 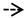 inflammation; 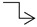 infiltration; 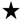 focal hepatic hemorrhage. CV: central vein. Different superscript letters (^a,b,c^) in the same row indicate significant differences at *p* < 0.05.

Representative views of liver sections are presented in Figure 9. As shown in tissue sections stained with hematoxylin and eosin, the liver tissues of normal rats exhibited the normal cellular structure with neat liver lobule, cords, and clear structure of portal area (Figure 9A). Compared with sections from livers in the control group, TEB presented morphological tissue degeneration, including intense cellular degeneration, sinusoidal dilatation, and severe inflammatory infiltration of hepatocytes with vascular congestion (Figure 9B). In addition, administration of TEB caused focal area of hepatic necrosis completely replaced by leucocyte cells infiltration and focal hepatic hemorrhage (Figure 9C). Compared to TEB-treated rats, oral administration of ACPs improved the hepatic architecture with the regeneration of liver parenchyma, as compared to TEB group (Figure 9D,E). ACPs group rats showed clearly less ballooning degenerative, less loose, less necrotic, and less inflammatory cell infiltration (Figure 9E). The histological results were consistent with biochemical data, which further confirmed the hepatoprotective effect of ACPs.

**Figure 10:**
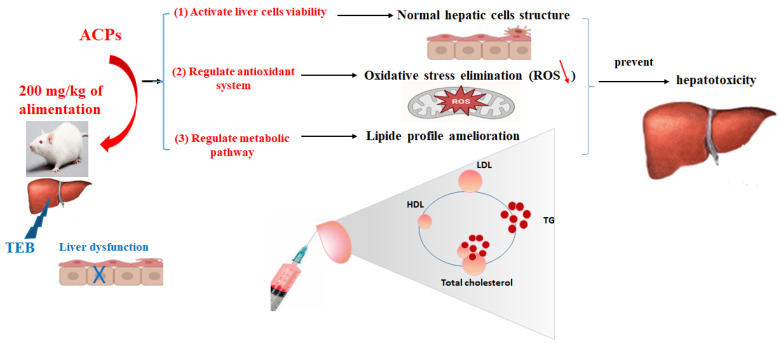
A proposed schematic diagram illustrating the protective mechanism of ACPs against TEB hepatotoxicity.

We summarized the current findings regarding the polysaccharides derived from the red macroalga *Alsidium corallinum* with hepatoprotective activity and described the underlying mechanisms (Figure 10). In general, the possible mechanisms of action by which polysaccharides exert their hepatoprotective activity are mainly divided into the following three directions: (1) ACPs activate the viability of hepatocyte cells by directly activating the protein responsible for cell differentiation; (2) ACPs can ameliorate the hepatic and serum index through oxidative stress response pathways by activating the antioxidant system; and (3) ACPs can balance the lipid profile by ameliorating cholesterol’s reverse transport and improving the anti-atherogenic metabolic pathway.

**Figure 11:**
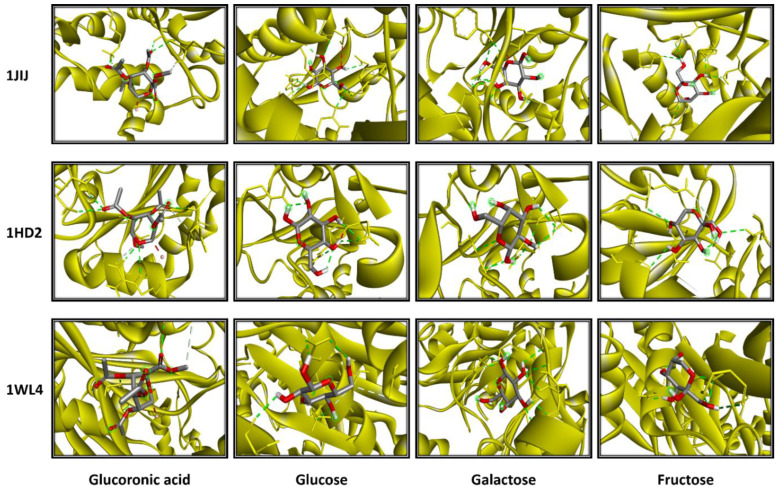
3D photographs of *Alsidium corallinum* monosaccharides complexed with the ribbon structure of 1JIJ, 1HD2, and 1WL4 receptors.

**Figure 12:**
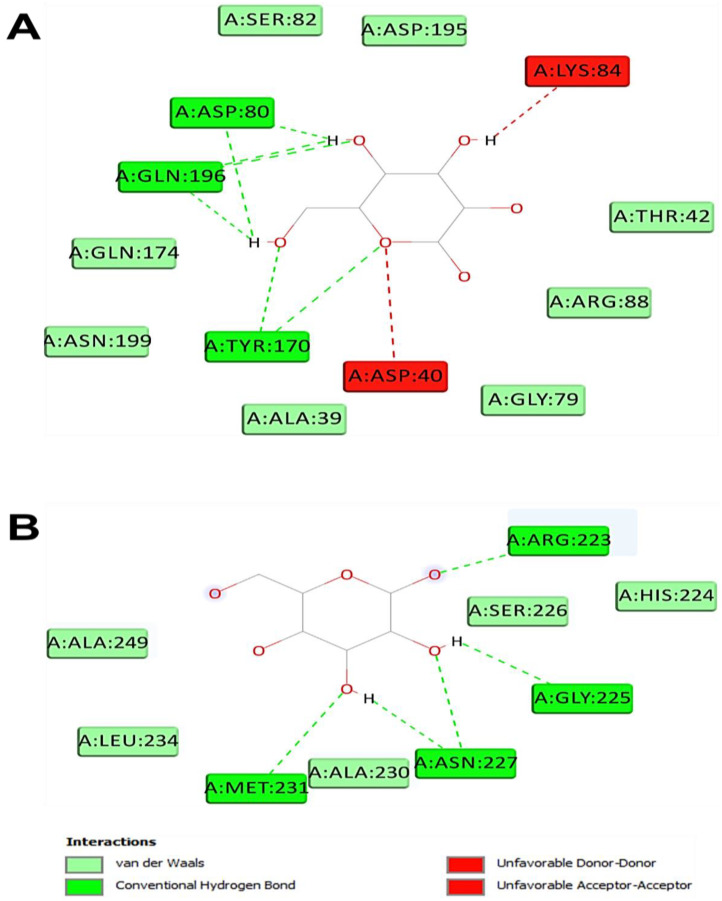
2D diagram of interactions of *Alsidium corallinum* monosaccharides. (**A**) Diagram of the interaction of galactose with 1JIJ, which possessed the best binding score (−7.1 kcal/mol). (**B**) Diagram of the interaction of galactose with 1WL4, which established the highest number of conventional H-bonds (n = 7).

The computational study exhibited that *A. corallinum* monosaccharides had various affinities to each targeted receptor (Table 5). Previous studies reported that variation in affinities is due to the chemical composition and geometry of the studied ligands [59,60]. Interestingly, all *A. corallium* monosaccharides possessed negative binding scores, which might contribute to their biological activities. These binding affinities varied between −5.8 and −7.1 kcal/mol for 1JIJ, between −5.1 and −5.4 kcal/mol for 1HD2, and between −4.9 and −5.2 kcal/mol for 1WL4. The best binding scores were predicted to galactose then to glucose while complexed to the TyrRS from *S. aureus* receptor, which may contribute to their highest biological activities, precisely the antimicrobial effect (Table 5 and Figure 11). Galactose established good molecular interactions with the targeted receptors that showed 5 or 7 conventional H-bonds supported with a network of carbon H-bonds, hydrophobic and/or electrostatic bonds, which significantly contribute to the stability of the complexes [61,62,63,64]. These molecular interactions included several key residues. They involved Gln196 (three times), Tyr170 (twice), and Asp80 and Asp80 once each for 1JIJ; and Asp77 (three times), Arg124 (twice), and Asn76, for 2XCT, and Asn227 (three times), Arg223 (twice), and Met231 and Gly225 once each for 1WL4 (Figure 11 and Figure 12).

The studied monosaccharides were also deeply embedded in the pocket region of 1JIJ, 1HD2, and 1WL4 receptors at a distance that reached 1.771 Å only between glucuronic acid and Ser87:HG of the Acyl-CoA: cholesterol acyltransferase. Overall, the calculated binding energies, the molecular interactions, and the deep embedding of *Alsidium corallinum* monosaccharides confirm that the potential antibacterial, antioxidant, and hypolipidemic effects are thermodynamically possible. The in vitro approaches and the in vivo results on TEB-intoxicated rats have already approved these effects. Our results also confirm the promising antimicrobial and antioxidant effects of medicinal plants and natural-derived compounds, including algae [61,62,63,64]. These effects may also include the acceptable bioavailability and the pharmacokinetic properties of the algal monosaccharides, which had been previously reported.

## 3. Materials and Methods

### 3.1. Source of Alga-Derived Polysaccharides

The marine red algae *Alsidium corallinum* was collected in the month of March from the coastal area of Sidi Mansour, Sfax, Tunisia. The voucher specimen of this species was deposited and identified in the herbarium of the Biology Laboratory at the Faculty of Sciences of Sfax. Alga was rinsed with tap water, followed by deionized water. Next, it was dried and ground before starting the extraction.

### 3.2. Extraction of Sulfated Polysaccharides (ACPs)

ACPs were extracted according to Chen’s method [65]. Briefly, *A. corallinum* powder was pre-extracted with ethanol (90%) to remove pigments. The dry residue was extracted twice with deionized water at 70 °C and stirred over six hours. The extract was combined and filtered, and the filtrate evaporated under a vacuum. The concentrated liquid was precipitated with ethanol for 24 h at 4 °C and then centrifuged for 15 min. The obtained residue was re-dissolved in double distilled water. The water phase was dialyzed at 4 °C against distilled water for 48 h. The dialysate was concentrated through rotary evaporation to obtain ACPs. The latter were stored at −20 °C for additional use.

### 3.3. Extraction Yield and Physicochemical Analysis of ACPs

ACPs extraction yield was measured based on the wet weight of *A. corallinum* powder. The yield of ACPs was expressed as a percentage (%) of the mass (g) of polysaccharides against the mass (g) of *A. corallinum* powder.

pH of ACPs (1% solution at 25 °C) was measured using a pH meter with complete immerging of the glass electrode into the solution.

The moisture and ash contents were determined at 105 and 550 ◦C, according to the AOAC standard methods 930.15 and 942.05, respectively [66].

The protein content was estimated by Bradford’s method [67]. Absorbance was measured by a spectrophotometer (595 nm). Protein levels were determined from a standard curve plotted using bovine serum albumin as a reference protein. All tests were carried out in triplicate.

Uronic acid amounts were determined by Blumenkrantz and Asbœ-Hansen’s method [68]. The absorbance was determined with a spectrophotometer (520 nm). The uronic acid amount was determined using a standard curve of glucuronic acid.

Carbohydrate amounts were determined by the method of Masuko [69]. Carbohydrate contents were measured by spectrophotometer at 490 nm. The result was estimated using glucose as a reference sugar from a standard curve.

### 3.4. Spectroscopic Analysis

#### 3.4.1. Monosaccharide Analysis by HPLC-FID

Various analytical methods and techniques are applied to evaluate the structure of the polysaccharide fragments. HPLC has been widely used to identify the constituent monosaccharides and molar ratios. The HPLC-FID assay was performed as described by Bayar et al. [70]. Monosaccharide composition was analyzed using a Sugar KS-800 column with a mobile phase of 0.001 M NaOH, flow rate of 0.5 mL/min, and column temperature of 60 °C. Fructose, arabinose, glucose, gluconic acid, sucrose, mannose, xylose, and galactose were used as standard.

#### 3.4.2. Fourier Transmission-Infra Red (FT-IR) Spectral Analysis

Infra-red spectroscopic analysis is a technique of measurement whereby spectra are collected based on the coherence of a radioactive source, using space-domain or time-domain measurements of electromagnetic radiation or other types of radiation. FT-IR spectroscopy investigates the vibrations of molecules and polar bonds between the different atoms. The FT-IR absorption bands were assigned according to previous literature observations to identify the chemical nature and functional structural groups [71]. The spectra obtained from the wave number 500–4000 cm^−1^ give some inside information about the tested compounds; therefore, the method is popular in identifying the vibrational structure of the materials [72]. Briefly, FT-IR spectrum of ACPs was determined on a Nicolet FT-IR spectrometer equipped with a horizontal attenuated total reflection (ATR) accessory. The internal crystal reflection was made from zinc selenide and had a 45° angle of incidence to the IR beam. The measurement was between 4000 and 500 cm^−1^. The transmission spectra of the samples were recorded by using the KBr pellets.

#### 3.4.3. Nuclear Magnetic Resonance (NMR) Spectroscopy

A powerful tool for the structural elucidation of polysaccharides is NMR spectroscopy, which can provide structural characteristics like monosaccharide components, anomeric configurations, sulfation linkages, and positions of branching [73]. Nuclear magnetic resonance is the only method that has the potential for full structural characterization of carbohydrates. This technique is seen as a screening test to determine the possible industrial value of raw extracts obtained from unexploited red algae [73]. Complete structural elucidation requires entirely assigning the ^1^H and ^13^C NMR spectra. Briefly, NMR spectrum of ACPs was obtained using an Avance DPX-500 NMR spectrometer (Bruker, Mannheim, Germany) at 500 MHz and 50 °C with acetone as the internal standard. The sample concentration was 10 mg of polysaccharides/mL of D2O for 1H and 13C.

### 3.5. In Vitro Biological Activities of ACPs

#### 3.5.1. DPPH Radical-Scavenging Assay

DPPH is an accurate, convenient, and quick method that analyzes the free radical scavenging ability of natural compounds in contrast to some other techniques [33]. DPPH assays can obtain the antioxidant competence of a compound. It is a synthetic free radical that antioxidants can effectively scavenge [34]. It has been used for rapid evaluation of the antioxidant ability of plant extracts. The DPPH of ACPs was performed according to the method described by Bersuder et al. [74]. The absorbance was measured at 517 nm, using a UV visible spectrophotometer. DPPH activity (I %) was calculated according to the equation:

I % = [(Abs control − Abs sample)/Abs control] × 100

where Abs control is the absorbance of the control reaction, and Abs sample is the absorbance of ACPs with the DPPH solution.

#### 3.5.2. ABTS Radical Scavenging Assay

ABTS radical scavenging activity is an exceptional method for testing the antioxidant activity of hydrogen-donating and chain-breaking antioxidants [75]. The ABTS was assayed according to Miller et al. method [76]. This method applies to hydrophilic and lipophilic antioxidants. A spectrophotometer measured the concentration of ABTS at 690 nm. The percentage inhibition of ACPs was calculated and compared with Trolox (6 hydroxy-2,5,7,8-tetramethylchroman-2-carboxylic acid) used as standard.

#### 3.5.3. Ferric Reducing Activity Power (FRAP)

The FRAP assay could estimate the capacity of antioxidants to reduce Fe^3+^-Fe^2+^ by providing an electron. The ability of the ACPs to reduce iron (III) was determined using the method of Fawole et al. [77] with some modifications. The resulting solution was measured at 700 nm. The reducing power was related to the absorbance of the reaction mixture. Vitamin C was used as a positive standard.

The reducing power was calculated according to the equation:

Reducing power = A_1_ − A_0_

#### 3.5.4. Nitric Oxide Radical Scavenging Activity

Nitric oxide radical scavenging activity of ACPs was determined using the method described by Marcocci et al. [78]. The solution was measured at 700 nm. The following formula calculated the result:

% Inhibition = (A control − A sample)/A control)) × 100

#### 3.5.5. In Vitro Evaluation of Antimicrobial Activity

Antimicrobial resistance may be acquired by genetic mutation in bacteria, making them resistant to the effect of one or more antimicrobial agents.

##### Microbial Strains and Growth Conditions

ACPs were assessed against a panel of microorganisms including six bacterial strains: Gram-negative: *Salmonella enterica*, *Escherichia coli*, and *Pseudomonas aeruginosa*, Gram-positive: *Micrococcus luteus*, *Bacillus amyloliquefaciens* and *Staphylococcus aureus*. All tested strains were obtained from the Microbiology laboratory, Faculty of Science of Sfax, Tunisia.

##### Disk Diffusion Method

For the antimicrobial activity, the disk diffusion method was employed, according to Nilsson et al. [79]. Antibacterial activity was estimated by measuring the diameters of the inhibition zones against the test organisms and compared to ampicillin and cycloheximide (10 µg per disk) as the positive control against bacteria yeast and fungi, respectively. The sliding caliper measured the accurate inhibition zone of any dimension surrounding the paper disk. This test was conducted in triplicate.

##### Microdilution Method

A broth microdilution method was used to resolve the minimum inhibitory concentration (MIC) and minimum bactericidal concentration (MBC) [80]. Ampicillin was used as a positive control against bacteria. The MIC is defined as the lowest concentration of the extract at which the microorganism does not reveal evident growth. The microorganism growth was estimated by the 3-(4,5-dimethylthiazol-2-yl)-2,5-diphenyltetrazolium bromide (MTT) assay, whereas the MBC expresses the lowest level of antimicrobial agent of the tested extract that results in microbial death. Each test was performed in three replicates.

### 3.6. ACPs Phytotoxicity Analysis

The phytotoxicity of the polysaccharides obtained from *A. corallinum* was evaluated by methods of Zucconi and Monaco [81] using the seed germination technique. Throughout the assay, control included only distilled water sterile. The length of seedling growths was measured as mm. The phytotoxicity of our polysaccharides was evaluated by percentage of germination index (GI %).

### 3.7. In Vivo Antioxidant Activities of ACPs

#### 3.7.1. Tebuconazole Presentation

Tebuconazole (TEB) is an effective fungicide triazole, with chemical formula C_16_H_22_C_l_N_3_O and >99% purity. TEB was provided by the Agriculture’s Company of Agrochemicals, Tunisia.

#### 3.7.2. Animal Diet and Tissue Preparation

Aged male rats (180 ± 20 g, n = 40) were divided into four groups, with 8 per group. The first group of rats served as the controls received no treatment (control group); the second group received 100 mg/kg body weight of TEB by intraperitoneal injection daily (TEB group); group III received TEB by the same dose and route as group II and 200 mg/kg of alimentation ACPs. Animals in the fourth group were given daily a single oral amount (200 mg/kg of diet) of ACPs. Doses of TEB and ACPs were selected based on previous studies [82,83] and checked before the experiment’s setting.

After 30 days of treatment, all animals were sacrificed. Liver tissue was divided into two parts: one for determining oxidative stress markers, and the other for the histological study. Blood was collected into heparinized tubes and centrifuged at 2200× *g* for 10 min. The plasma samples were stored at −80 °C until biochemical analysis. The experimental procedures of animals were performed according to the Natural Health Institute of Health Guidelines for Animal Care approved by the Institute Ethical Committee Guidelines Council of European Communities [84] and the use of laboratory animals of our institution.

#### 3.7.3. Liver Protein Quantification

Lowry et al. [85] measured liver protein contents with bovine serum albumin as standard.

#### 3.7.4. Determination of Oxidative Stress Markers

Lipid peroxidation in the liver tissue was determined according to the Draper and Hadley method [86]. The amount of malondialdehyde (MDA) was estimated colorimetrically by measuring thiobarbituric acid reactive substances (TBARS), which were expressed in terms of malondialdehyde content. The TBARS were determined by reading absorbance at 532 nm. The result was expressed as µmol /mg protein.

The hydrogen peroxide (H_2_O_2_) test used the ferrous ion oxidation xylenol orange method [87]. The FOX1 reagent consisted of 25 mM sulfuric acid, 250 mM ferrous ammonium sulfate, 100 mM xylenol orange, and 0.1 M sorbitol. Briefly, 100 mL of extract was added to 900 mL of FOX1 reagent vortexed and incubated for 30 min at room temperature. Solutions were then centrifuged at 12.000× *g* for 10 min, and the amount of H_2_O_2_ in the supernatant was determined using a spectrophotometer at 560 nm. H_2_O_2_ levels were expressed as μmol/mg protein.

Advanced oxidation protein product (AOPP) concentrations in liver tissue were analyzed using the method used by Witko et al. [88]. Briefly, 0.4 mL of liver extract was treated with 0.8 mL phosphate buffer (0.1 M; pH 7.4). After 2 min, 0.1 mL 1.16 M potassium iodide (KI) was added to the tube, followed by 0.2 mL of acetic acid. The absorbance of the reaction mixture was immediately recorded at 340 nm. The concentration of AOPP for each sample was calculated using the extinction coefficient of 26.1 cm^−1^ mmol^−1^, and the results were expressed as nmol/mg protein.

Superoxide dismutase (SOD) enzyme activity was assayed using the method of Beauchamp and Fridovich [89]. The reaction mixture contained 50 mL of the liver homogenate in Tris HCl buffer (pH 7.8), 13 mM Lmethionine, 75 mM Nitro Blue Tetrazolium (NBT), 0.1 mM EDTA, and 2 mM riboflavin. The developed blue color of the reaction was measured at 560 nm. The limit of detection of the method is 0.195 units. Units of SOD activity were expressed as the amount of enzyme required to inhibit the reduction in NBT by 50% and the activity was expressed as units/mg of protein.

Glutathione peroxidase (GPx) enzyme activity was assayed according to the method described by Flohe and Gunzler [90]. GPx catalyzes the oxidation of reduced glutathione by cumene hydroperoxide. In the presence of nicotinamide adenine dinucleotide phosphate reduced form (NADPH) and reduced glutathione reductase, the disulfide reduced glutathione is immediately converted to the reduced form with concomitant oxidation of NADPH. Briefly, 200 mL of the homogenized tissue was added to 200 mL of the reduced glutathione reductase (4 mM) and 100 mL of 100 mM phosphate buffer, pH 7.4. In the presence of nicotinamide adenine dinucleotide phosphate reduced form (NADPH), the oxidized, reduced glutathione is immediately converted to the reduced form with concomitant oxidation of NADPH/NADPþ. The absorbance was measured at 340 nm. The results were expressed as nmol glutathione oxidized/min/mg protein.

Glutathione (GSH) levels were estimated by Ellman [91] and modified by Jollow et al. [92]. The method is based on developing a yellow color when DTNB (5,5′-dithiobis-2-nitrobenzoic acid) was added to compounds containing sulfhydryl groups. In brief, five hundred microliters of liver homogenate in Tris Hcl buffer were added to 3 mL of 4% sulfosalicylic acid. The mixture was centrifuged at 3500× *g* for 10 min. Ellman’s reagent was added to five hundred microliters of supernatants. The absorbance was measured at 412 nm after 10 min. The absorbance was recorded at 412 nm after 10 min. Total GSH content was expressed as nmol/mg of protein.

#### 3.7.5. Biochemical Index Measurements

Plasma aspartate aminotransferase (AST) and alanine aminotransferase (ALT) activities and bilirubin levels, which are considered to be the biochemical markers of hepatic damage, were assayed according to the standard procedures using the commercial kits purchased from Biomaghreb (Ariana, Tunisia, References: 20043; 20047 and 20102, respectively) [16].

#### 3.7.6. Lipid Profile in Plasma

Total cholesterol, HDL-cholesterol, and triglycerides levels were determined using kits obtained from Biomaghreb (Tunisia, References 20111, 20113, and 20131, respectively) [16]. The low-density lipoprotein cholesterol (LDL-C) fraction was determined using the Friedewald equations:

[LDL − cholesterol] = Total cholesterol − [(Triglyceride/5) + HDL-cholesterol].

#### 3.7.7. Histological Examination

The liver collected from each group was randomly selected for light microscopy. Samples were fixed in a formalin solution and embedded in paraffin. They were then sectioned and stained with hematoxylin-eosin. All liver tissue sections were evaluated for the degree of injury [93].

### 3.8. Computational Analysis and Interactions Assay

The four monosaccharides in *Alsidium corallinum*, which had been identified, were used in the computational study to decipher their molecular interactions and confirm their potential antioxidant, antimicrobial, and hypolipidemic effects. The chemical structure of these monosaccharides was collected from the Pubchem website. The 3D crystal structure of TyrRS from *Staphylococcus aureus* (1JIJ), human peroxiredoxin (1HD2), and Acyl-CoA: cholesterol acyltransferase (ACAT, 1WL4) were obtained from the RCSB PDB. The studied monosaccharides and three targeted receptors were prepared, processed for depreciation, and then saved in pdbqt format [61,63]. As previously reported, they have been subjected to a CHARMm force field after targeting the grid box by selecting some key residues within the pocket region [57,58,59,60]. The reasons behind choosing these receptors are the massive responsibility of hospital-acquired infections by *S. aureus*, the key role of reducing hydrogen peroxide and alkyl hydroperoxides using equivalents, and the key involvement in fatty acid metabolism.

### 3.9. Statistical Analyses

All experiences and statistical analyses were made in triplicate. All results are expressed as the mean standard deviation. Statistical analysis was performed with the SPSS 17.0 statistical package for Windows (SPSS, Inc., Chicago, IL, USA). A two-way ANOVA followed by Tukey’s post hoc test was performed to compare treatment and control groups. Statistical significance was set at *p* = 0.05.

## 4. Conclusions

The polysaccharides from *A. corallinum* exhibited protective effects against TEB-induced toxicity by reducing ROS production. Both finding scores and molecular interactions of *A. corallinum* polysaccharides may explain the experimental in vitro and in vivo findings, which may result in the antioxidant, antimicrobial, and hypolipidemic activities. Further studies are required to clarify the mechanism of action of algae polysaccharides on liver cells and to probe the clinical availability of these compounds in the form of algal foods, food supplements, and regulated therapeutics.

## Figures and Tables

**Table 1:** Physicochemical properties of ACPs.

Parameters	ACPs
Yield (%)	12.63 ± 1.39
pH	6.20 ± 0.20
Moisture (%)	2.96 ± 0.09
Ash (%)	3.00 ± 0.08
Proteins (%)	1.94 ± 0.07
Uronic acid (%)	12.06 ± 1.74
Carbohydrates (%)	66.06 ± 4.69

Values are given as the mean of three determinations (X ± SD); SD: standard deviation.

**Table 2:** Antimicrobial activity of ACPs.

Microorganisms	DD (mm) ACPs	DD (10 μg/disk)	MIC (µg/mL)	MBC (µg/mL)	R
*E. coli*	9.66 ± 0.50	28	100	50	2
*S. enterica*	9.33 ± 0.70	21	100	100	1
*P. aeruginosa*	9.55 ± 1.00	39	100	100	1
*M. luteus*	9.66 ± 0.50	15	100	100	1
*L. invanovii*	9.00 ± 1.00	25	100	100	1
*S. aureus*	10.66 ± 0.10	23	50	100	2

**Table 3:** Effect of ACPs on toxicity biomarkers in hepatic tissue of TEB treated rats.

Parameters and Treatments	Control	TEB	TEB + ACPs	ACPs
MDA (µmol of MDA/mg protein)	90.86 ± 16.47 ^a^	187.24 ± 6.10 ^c^	148.79 ± 11.54 ^b^	114.82 ± 15.83 ^a^
H_2_O_2_ (µmol/mg protein)	0.07 ± 0.01	0.12 ± 0.01 ^c^	0.09 ± 0.03 ^b^	0.08 ± 0.02 ^a^
AOPPs (nmol/mg protein)	0.59 ± 0.07 ^a^	0.86 ± 0.08 ^b^	0.77 ± 0.06 ^b^	0.63 ± 0.1 ^a^

Values are expressed as means ± S.D for 6 animals in each group. Different superscript letters (^a,b,c^) in the same row indicate significant differences at *p* < 0.05.

**Table 4:** Effect of ACPs on antioxidant defense in hepatic tissue of TEB treated rats.

Parameters and Treatments	Control	TEB	TEB + ACPs	ACPs
GPx (nmol GSH/ min/mg protein)	6.11 ± 1.13 ^b^	3.60 ± 0.59 ^a^	4.65 ± 0.97 ^ab^	5.64 ± 0.39 ^b^
SOD (units/mg protein)	23.34 ± 4.22 ^b^	12.76 ± 4.17 ^b^	18.43 ± 2.11 ^b^	25.62 ± 2.62 ^b^
GSH (nmol/mg protein)	20.35 ± 4.65 ^b^	11.38 ± 3.13 ^b^	14.35 ± 3.54 ^b^	18.08 ± 2.56 ^b^

Values are expressed as means ± S.D for six animals in each group. Different superscript letters (^a,b^) in the same row indicate significant differences at *p* < 0.05.

**Table 5:** Binding affinity, conventional hydrogen bonds, and interacting residues of *Alsidium corallinum* monosaccharides complexed with 1JIJ, 1HD2, and 1WL4 receptors.

Monosaccharide (Ligand)	Intermolecular Interactions			
	Binding Affinity (kcal/mol)	No. H-Bond	Closest Interacting Residues (Distance, Å)	Closest Interacting Residue
TyrRS from *S. aureus* (1JIJ)				
Glucuronic acid	−5.8	4	Lys226 (2.155), Lys226 (2.112), Gly233 (2.446), Lys234 (2.721), Lys234 (3.468)	Lys226:HZ3
Glucose	−7.0	8	Asn124 (2.835), Gln174 (2.131), Asp80 (2.449), Gln196 (2.745), Asp40 (2.216), Tyr36 (2.241), Gln190 (2.511), Asp177 (1.898)	Asp177:OD1
Galactose	−7.1	7	Tyr170 (2.845), Tyr170 (2.678), Gln196 (2.478), Asp80 (2.185), Gln196 (2.197), Asp80 (2.039), Gln196 (2.565)	Asp80:OD2
Fructose	−6.6	5	Asp80 (2.752), Gln196 (2.824), Thr75 (2.171), Tyr170 (2.402), Tyr36 (2.647)	Tyr75:OG1
Human peroxiredoxin (1HD2)				
Glucuronic acid	−5.3	6	Asn21 (2.581), Asn21 (2.362), Arg86 (3.037), Arg86 (2.250), Gly92 (2.173), Leu96 (2.735), Gly82 (3.541), Glu91 (3.550)	Gly92:HN
Glucose	−5.4	4	Asn76 (2.064), Asn122 (2.303), Asp77 (2.368), Asp77 (2.432)	Asn76:HD21
Galactose	−5.1	5	Asn76 (2.077), Asp77 (2.309), Arg124 (2.861), Arg124 (2.431), Asp77 (2.579), Asp77(2.488)	Asn76:HD21
Fructose	−5.3	5	Gly17 (3.034), Gly92 (2.823), Val94 (2.584), Thr81 (2.738), Leu96 (2.998), Glu16 (3.557)	Val94:O
Acyl-CoA: cholesterol acyltransferase (ACAT, 1WL4)				
Glucuronic acid	−5.2	3	Asn68 (2.254), Ser87 (2.125), Ser87 (1.771), Gly66 (3.694)	Ser87:HG
Glucose	−4.9	5	Thr36 (2.454), Asp32 (2.429), Asp32 (2.495), Asp32 (2.468), Leu206 (2.527), Ser35 (3.564), Gly76 (3.650)	Asp32:OD1
Galactose	−4.9	7	Arg223 (2.284), Arg223 (2.036), Asn227 (2.563), Asn227 (2.688), Met231 (2.864), Asn227 (2.369), Gly225 (2.553)	Arg223:HH21
Fructose	−4.9	5	Ser208 (1.870), Ser208 (2.117), Ser208 (1.880), Ser208 (3.072), Asp32 (1.916)	Ser208:HN

## Data Availability

Data is contained within the article.
